# Potential prognostic value of delta-like protein 3 in small cell lung cancer: a meta-analysis

**DOI:** 10.1186/s12957-020-02004-5

**Published:** 2020-08-26

**Authors:** Benchao Chen, Heng Li, Chao Liu, Shuting Wang, Feiyue Zhang, Li Zhang, Mingjie Li, Gaofeng Li

**Affiliations:** Department of Thoracic Surgery, The Third Affiliated Hospital of Kunming Medical University (Yunnan Cancer Hospital), Kunming, Yunnan China

**Keywords:** Delta-like protein 3, DLL3, Prognostic, Small cell lung cancer, SCLC, Meta-analysis

## Abstract

**Background:**

Current researches have revealed that delta-like protein 3 (DLL3) may be related with prognosis in patients with small cell lung cancer (SCLC). However, this finding remains controversial in small cell lung cancer. This meta-analysis was systematically performed to evaluate the prognostic value of DLL3 in SCLC.

**Methods:**

The PubMed, EMBASE and Web of Science databases were retrieved to collect the eligible references. Through Stata 15.0 software, we pooled hazard ratios (HR) with 95% confidence intervals (CI) by using random or fixed-effects models to evaluate the association between DLL3 and SCLC survival results.

**Results:**

A total of 6 inter-related studies including 645 patients were qualified. After we removed 1 study, the remaining 5 studies including 601 patients were pooled to testify that high expression of DLL3 was an inferior prognostic for patients with SCLC in Asian populations (HR = 1.37, 95% CI = 1.05, 1.69; *I*^2^ = 0.0%, *p* = 0.000). The pooled results showed that DLL3 might be higher expression in advanced metastasis SCLC in Asian populations (RR = 0.84, 95% CI = 0.71, 0.99; *I*^2^ = 44.7%, *p* = 0.039). But the expression of DLL3 was not correlated with sex (RR = 1.33, 95% CI = 0.98, 1.80; *I*^2^ = 0.0%, *p* = 0.064), smoking history (RR = 1.01, 95% CI = 0.58, 1.75; *I*^2^ = 72.1%, *p* = 0.967) and tumour stage (RR = 0.68, 95% CI = 0.44, 1.05; *I*^2^ = 66.6%, *p* = 0.081).

**Conclusions:**

Our meta-analysis confirms that in Asian populations, high expression of DLL3 was a potential poor prognostic biomarker for SCLC and DLL3 highly expressed in advanced stage SCLC in Asian populations.

## Introduction

Lung cancer has the highest morbidity and mortality of all malignant tumours, which is one of the most common cancers worldwide [1]. The major histologic subtypes of lung cancer are non-small cell lung cancer (NSCLC, 85%) and small cell lung cancer (SCLC, 15%) [2]. SCLC is an invasive, high-grade malignancy with early development and a bad prognosis. Most patients with SCLC are advanced with widespread metastasis when they are diagnosed, and systemic chemotherapy is the most effective therapy [3]. Few therapeutic methods are available after SCLC relapse, and the prognosis is especially poor. Therapeutic treatments for patients with SCLC have essentially remained unchanged in recent years [4]. Thus, further research into the mechanism of SCLC and the exploration of new therapeutic targets for SCLC is imperative.

Delta-like protein 3 (DLL3) is a transmembrane protein that promotes the development of neuroendocrine tumours through its reciprocity with the Notch pathway [5]. It is expressed in cancer tissues in approximately 85% of patients with SCLC and other neuroendocrine cancer but is not expressed in non-neuroendocrine tumours or normal tissues [6]. Most studies have shown that DLL3 is a latent treatment target point for SCLC [7–9]. Recently, many researches initiate to focus on the prognostic value of DLL3 in SCLC. Some of the researchers demonstrated that DLL3 had no correlation with prognosis in SCLC patients [10, 11], but other investigators showed that DLL3 was associated with the survival of patients with SCLC [12]. So previous studies of the prognostic value of DLL3 in SCLC remain controversial, we performed this systematic meta-analysis for resolving this controversial.

Published articles discussing the prognostic value of DLL3 in SCLC were systematically reviewed in our meta-analysis. We aimed to include all correlational studies to assess the prognostic value of DLL3 and attempted to identify an accurate biomarker to guide prognosis and treatment for SCLC in the future.

## Methods

### Search strategy

The PRISMA guidelines were followed for our meta-analysis (see Additional file 1) [13]. The literatures were collected through retrieving the PubMed, EMBASE and Web of Science databases from the initial date to February 25, 2020. The search strategy was “DLL3 OR delta-like protein 3” (all fields) AND “non-small cell lung cancer OR NSCLC OR small cell lung cancer OR SCLC OR Lung Adenocarcinoma OR Lung Squamous cell carcinoma OR lung cancer OR lung tumour OR lung neoplasm OR lung carcinoma” (all fields). There was no limitation on the publication status. All eligible studies were retrieved, and the headlines and abstracts of all the reference lists of the reviews or studies were independently filtrated through three authors based on the criteria. Differences between two authors were resolved by the third author’s opinions.

### Inclusion criteria

Researches were included by following the standards: (1) studies reported the prognostic value of DLL3 in SCLC, (2) studies were published as original articles, (3) studies reported the data of HR and 95% CI or provided survival curve and (4) studies in which the prognostic value was investigated by survival analysis with overall survival (OS), disease-free survival (DFS), progression-free survival (PFS), relapse/recurrence-free survival (RFS) or disease-specific survival (DSS). We excluded the animal researches and other review literatures.

### Data extraction

Two researchers independently extracted the data included the author, country, edition year, sample capacity, patients’ sex, smoking history, distant metastasis, tumour stage, histologic subtype, biomarker(s), scoring methods, cut-offs value and the data of HR and 95% CI. We extracted the multivariate analysis outcome if one study included both univariate and multivariate analysis outcomes. When we could not extract the data of HR and 95% CI directly in the article but the survival curves were reported, we extracted and digitized the survival curves by operating the Engauge Digitizer software (http://digitizer.sourceforge.net/), and we estimated the data of HR and 95% CI by the Excel programme files as exploited by Tierney et al. [14]. When the data of HR and survival curves were not reported, we contacted the corresponding authors of eligible articles by email to obtain the original data; these articles were excluded if there was no response.

### Assessment of study quality

Two researchers independently used the Quality In Prognosis Studies (QUIPS) tool to assess risk of bias of all the publications [15]. According to the criteria, every article was evaluated as low risk, moderate risk or high risk by following six different areas: study participation, study attrition, prognostic factor measurement, outcome measurement, study confounding and statistical analysis and reporting [15]. Differences were settled through discussion.

### Statistical analysis

Two authors independently extracted the HR and 95% CI from the original articles. The HR was used to describe the high DLL3 expression versus low DLL3 expression. We took the reciprocal of the HR if the articles showed the low DLL3 expression vs. high DLL3 expression. We observed that high expression of DLL3 portended a worse prognosis when HR > 1 and that HR < 1 indicated preferable prognosis. For the analysis results, *p* < 0.05 was considered statistically significant. Statistical heterogeneity was assessed by calculating the *I*^2^ statistic [16]. The presence of heterogeneity was indicated when *I*^2^ > 50%, and then a random-effects model was applied to pool the results [17]; a fixed-effects model was employed to pool the results when *I*^2^ < 50% [18]. We performed further subgroup or sensitivity analysis to analyse the heterogeneity. The publication bias was estimated by Begg’s and Egger’s tests, when *p* > 0.05 indicates no publication bias [19]. Through Stata 15.0 software, we performed this meta-analysis and acquired the forest plots.

## Results

### Search results and study characteristics

**Fig. 1:**
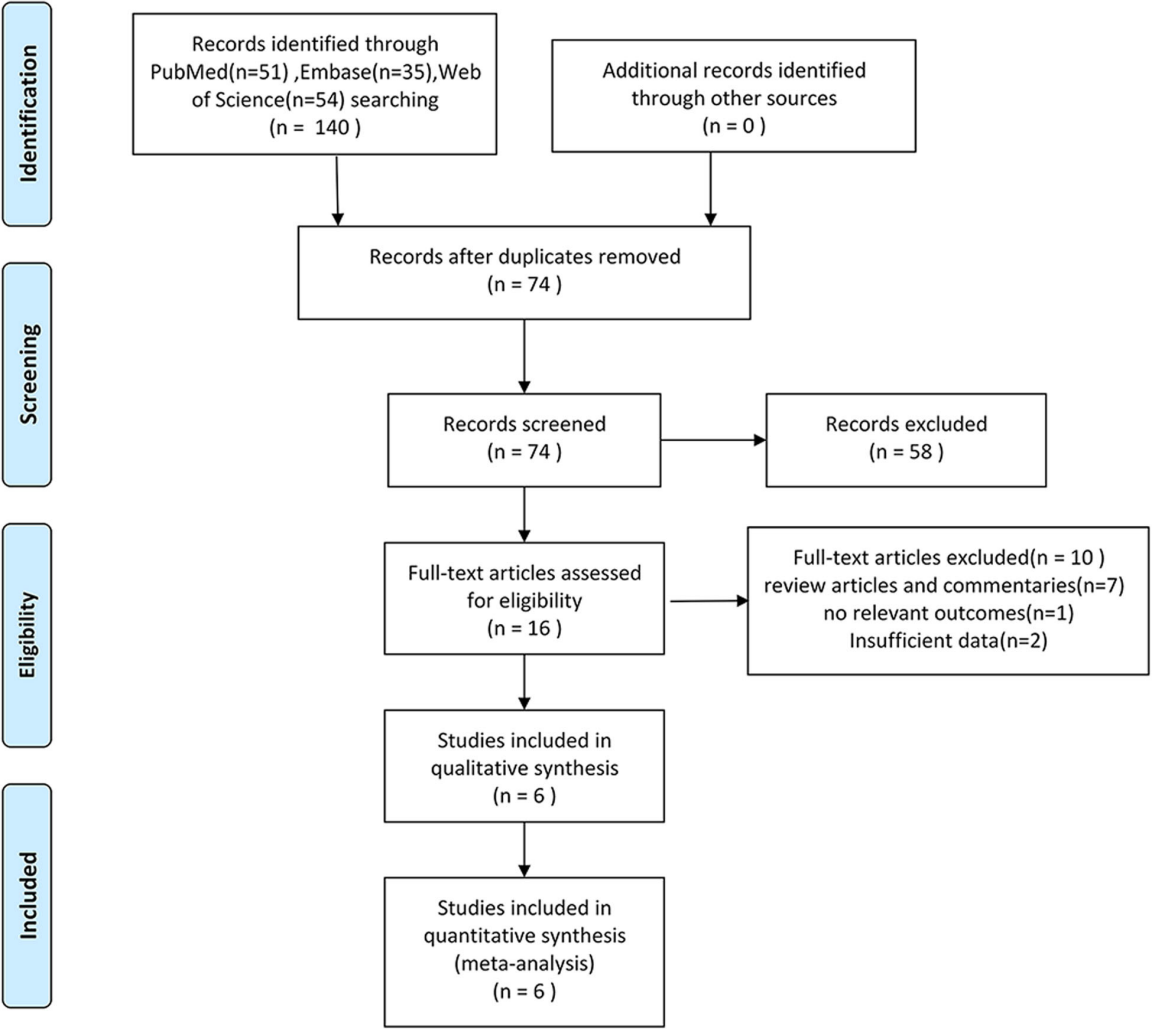
The flow diagram of studies selection

Using the searching strategy described above, a total of 140 original documents were identified from the databases, with approximately 74 studies remaining after excluding duplicates. After screening the titles and abstracts of the 74 publications, 58 publications were not related to evaluating the prognosis role of DLL3 in SCLC. Finally, we were left with 16 eligible studies after screening the full text, among which 6 articles were included in our final analysis (Fig. 1) [20–25]. Ten articles were excluded for the following reasons: seven articles were review researches and commentaries, one article did not report hazard ratios and Kaplan-Meier curves and 2 articles were abstracts and did not report relevant outcomes. The 6 eligible articles’ characteristics are shown in Table 1. All these articles came out from the initial date to February 25, 2020, and the patients were diagnosed as SCLC by histopathology. Among these studies, all studies investigated DLL3 by immunohistochemistry (IHC) in paraffin-embedded tissue.

**Table 1:** Study characteristics of the 6 eligible articles

Study	Year	Country	No. of samples	Sex (male/female)	Smoking history (non/yes)	Distant metastasis (negative/positive)	Tumour stage (I-II/III-IV)	Histologic subtype	Method	Outcomes	Biomarkers	Cut-off value
Yan et al. [20]	2019	China	335	306/29	93/242	165/170	29/306	SCLC	IHC	OS	DLL3/TTF1	score ≥ 150
Xie et al. [21]	2019	USA	44	19/25	N/A	N/A	19/16	SCLC	IHC	OS	DLL3	≥ 50%
Tanaka et al. [22]	2018	Japan	63	52/11	3/60	N/A	N/A	SCLC	IHC	OS	DLL3	≥ 50%
Regzedmaa et al. [23]	2019	China	38	26/12	12/26	15/23	16/22	SCLC	IHC	OS	DLL3/CTLA-4/MSTN	score ≥ 13.5
Huang et al. [24]	2019	China	72	N/A	N/A	N/A	N/A	SCLC	IHC	OS/PFS	DLL3	score ≥ 6
Furuta et al. [25]	2019	Japan	93	72/21	18/75	N/A	82/11	SCLC	IHC	OS	DLL3/ASCL1	≥ 75%

*SCLC* small cell lung cancer, *IHC* immunohistochemistry, *OS* overall survival, *PFS* progression-free survival, *DLL3* delta-like protein 3, *CTLA-4* cytotoxic T lymphocyte-associated protein 4, *MSTN* mesothelin, *ASCL1* achaete-scute homologue-1, *N/A* not applicable

### Quality assessment of the included studies

We performed quality evaluations of the 6 articles following the QUIPS tool, and two authors independently evaluated all the literature. Differences were resolved by discussion. After screening all included articles, we found that there were no researches that reported study attrition, and there were no standardization of cut-offs value for evaluating DLL3 expression. And then 3 studies were evaluated as low risk, 2 were evaluated as moderate risk and 1 study was evaluated as high risk (Table 2). This outcome indicated that most of the studies we included were of a medium or high quality.

**Table 2:** Quality assessment of included studies

Study	Study participation	Study attrition	Prognostic factor measurement	Outcome measurement	Study confounding	Statistical analysis and reporting	Total risk of bias
Yan et al. [20]	○	●	○	○	○	○	Low
Xie et al. [21]		●	○	○		○	Moderate
Tanaka et al. [22]	○	●	○	○	○	○	Low
Regzedmaa et al. [23]	○	●	○	○	○	○	Low
Huang et al. [24]	○	●	○		●	○	High
Furuta et al. [25]		●	○	○		○	Moderate

“◎” indicates low risk of bias, “ ” moderate risk of bias and “●” high risk of bias

### Prognostic value of DLL3 in SCLC

**Fig. 2:**
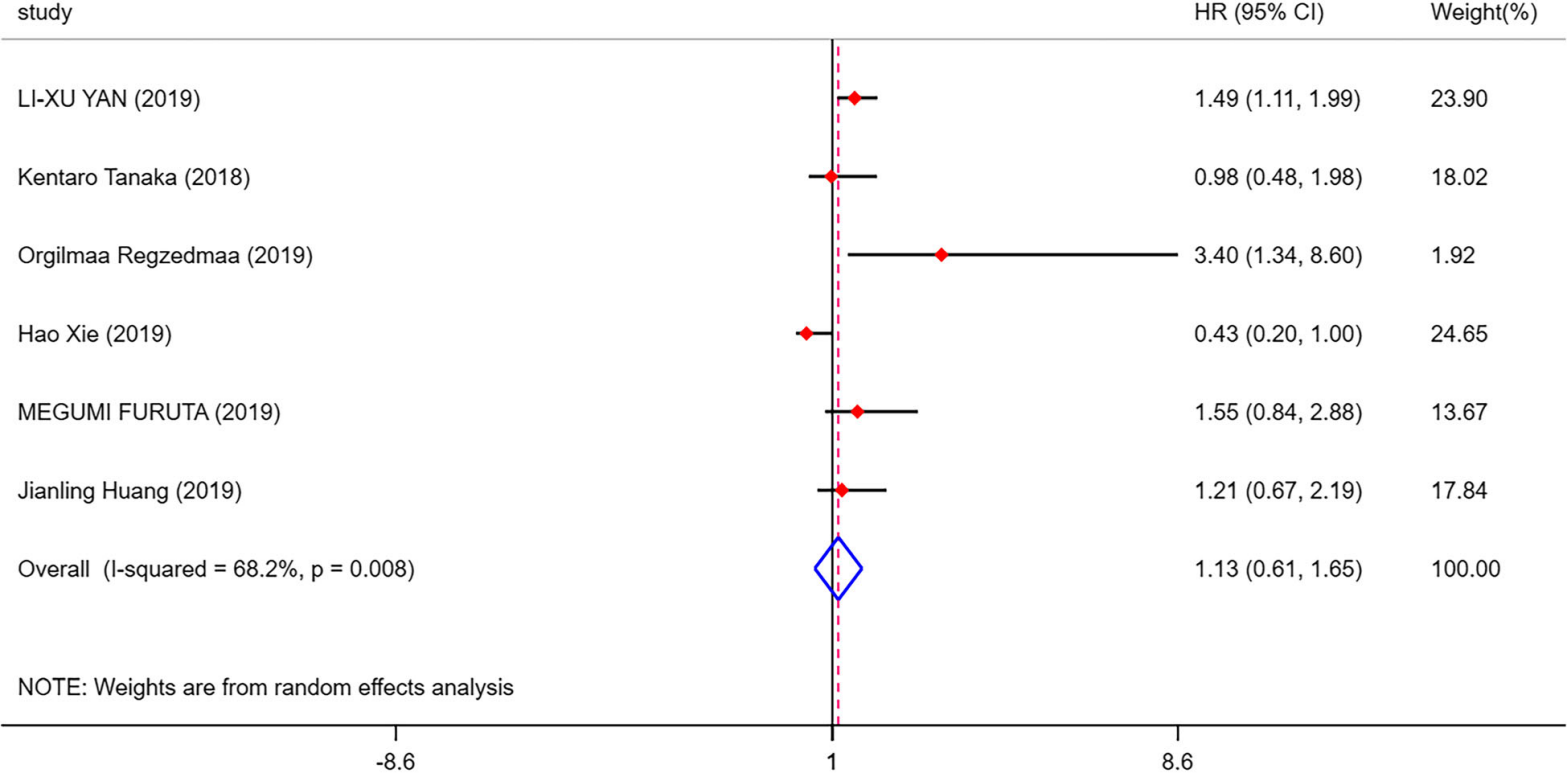
Forest plots of prognostic value of DLL3 in SCLC patients. DLL3, delta-like protein 3; HR, hazard ratios; 95% CI, 95% confidence intervals; I-squared, percentage heterogeneity between studies; *p,* test for heterogeneity

**Fig. 3:**
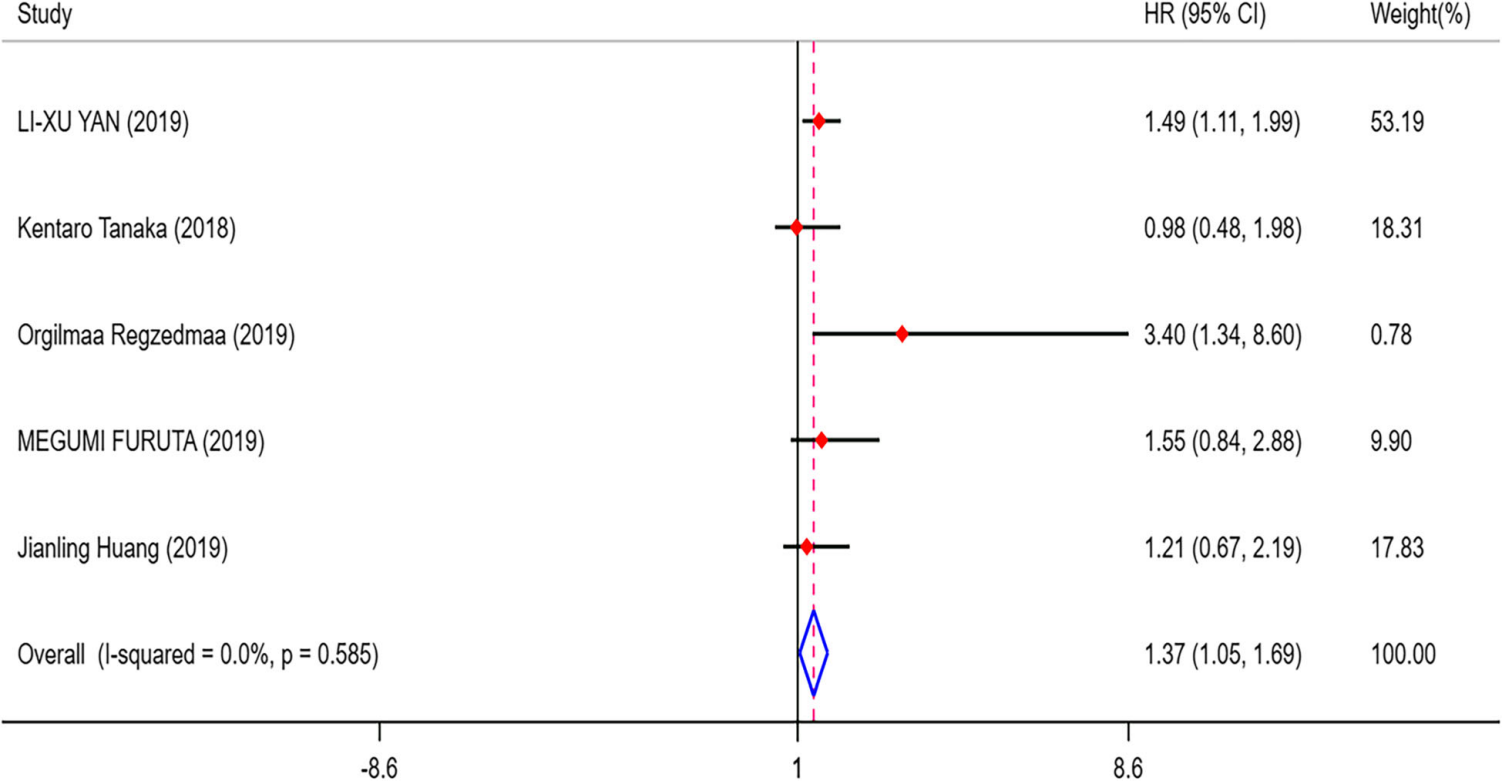
Forest plots of prognostic value of DLL3 in Asia patients with SCLC.DLL3, delta-like protein 3; USA, the United States of America; HR, hazard ratios; 95% CI, 95% confidence intervals; I-squared, percentage heterogeneity between studies; *p,* test for heterogeneity

**Fig. 4:**
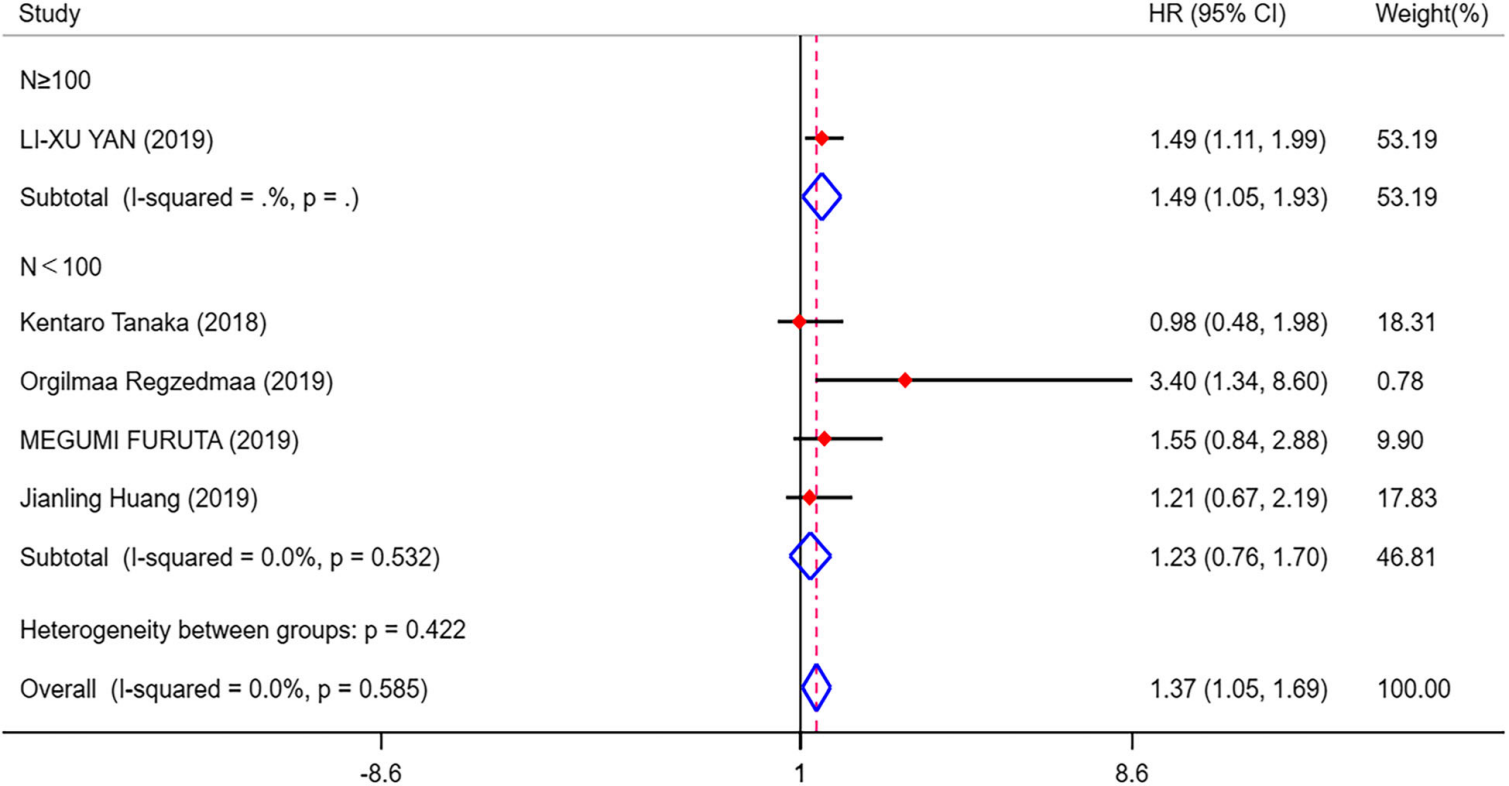
Forest plots of prognostic value of DLL3 in different studies with different sample sizes. DLL3, delta-like protein 3; HR, hazard ratios; 95% CI, 95% confidence intervals; I-squared, percentage heterogeneity between studies; *p,* test for heterogeneity

**Fig. 5:**
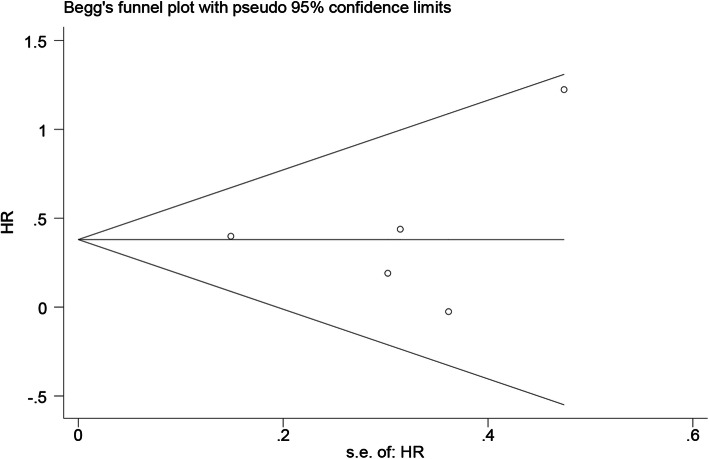
Funnel plot of DLL3 present on overall survival. DLL3, delta-like protein 3; HR, hazard ratios

As shown in Fig. 2, 6 eligible articles were pooled for analysing the prognostic value of DLL3 in SCLC. The results showed that high expression of DLL3 indicated no prognostic value in patients with SCLC (HR = 1.13, 95% CI = 0.61, 1.65; *I*^2^ = 68.2%, *p* = 0.008). However, prominent heterogeneity existed in the pooled results, so we conducted further subgroup analysis. Among the six studies, three articles were from China, two were from Japan and one was from America. We divided the studies into the Asian group and the American group for subgroup analysis. As shown in Additional file 2. Fig.A.1, we found that the heterogeneity was 0.0% in the Asian group, and we believed that the main cause of the heterogeneity was the study from American. So we decided to eliminate the study from American and only analysed the remained 5 studies. After we removed the American study, the outcomes indicated that high DLL3 expression was a poor prognosis marker in SCLC (HR = 1.37, 95% CI = 1.05, 1.69; *I*^2^ = 0.0%, *p* = 0.000) (Fig. 3). Then, we divided the 5 studies into two groups according to the size of the sample, and the final outcomes indicated that high DLL3 expression was a poor prognosis marker in the group with sample size greater than 100 (HR = 1.49, 95% CI = 1.05, 1.93, *p* = 0.000) (Fig. 4). But there was no significant relationship between DLL3 expression and prognosis in the group with sample size less than 100 (HR = 0.99, 95% CI = 0.45, 1.54; *I*^2^ = 53.7%, *p* = 0.000) (Fig. 4). Begg’s test (*p* = 0.624) and Egger’s test (*p* = 0.103) results demonstrated that there was no publication bias in our meta-analysis (Fig. 5). Finally, sensitivity analysis showed that all the studies were stable (see Additional file 2. Fig.A.6).

### The clinical characteristics of patients with DLL3 expression in SCLC

The pooled results showed that the expression of DLL3 was not correlated with sex (RR = 1.33, 95% CI = 0.98, 1.80; *I*^2^ = 0.0%, *p* = 0.064) (Table 3, Additional file 2. Fig.A.2), smoking history (RR = 1.01, 95% CI = 0.58, 1.75; *I*^2^ = 72.1%, *p* = 0.967) (Table 3, Additional file 2. Fig.A.3) and tumour stage (RR = 0.68, 95% CI = 0.44, 1.05; *I*^2^ = 66.6%, *p* = 0.081) (Table 3, Additional file 2. Fig.A.4). But DLL3 was highly expressed in patients with distant metastasis (RR = 0.84, 95% CI = 0.71, 0.99; *I*^2^ = 44.7%, *p* = 0.039) (Table 3, Additional file 2. Fig.A.5).

**Table 3:** Summary of the correlation between DLL3 expression and the clinical characteristics of patients with SCLC

Clinical characteristics	No. of studies	*I*^2^ (*p*)	RR (95% CI)	*P* value
Sex (male/female)	3	0.0% (0.798)	1.33 (0.98, 1.80)	0.064
Smoking history(non/yes)	3	72.1% (0.028)	1.01 (0.58, 1.75)	0.967
Distant metastasis (negative/positive)	2	44.7% (0.179)	0.84 (0.71, 0.99)	0.039
Tumour stage (I-II/III-IV)	3	66.6% (0.050)	0.68 (0.44, 1.05)	0.081

*SCLC* small cell lung cancer, *RR* relative ratios, *95% CI* 95% confidence intervals, *DLL3* delta-like protein 3, *I*^*2*^ percentage heterogeneity between studies, *p* test for heterogeneity

## Discussion

Many studies have shown that DLL3 played a significant prognosis value in patients with cancer. For example, high DLL3 expression was associated with shorter OS and PFS in small cell bladder cancer [26]. High DLL3 expression was associated with bad OS and usually expressed in older patients and advanced stage in endometrial cancer [27]. Xie et al. reported that high DLL3 expression predicted a better OS in patients with SCLC [21], and other studies foreboded that high DLL3 expression was an inferior prognostic marker for SCLC [20, 23]. Thus, the results of different studies remained controversial. We included a total of 5 studies with 601 patients with SCLC to assess the prognostic value of DLL3 in SCLC by pooling the data of HR and 95% CI.

First, we performed this meta-analysis using 6 studies. But heterogeneity was observed in our pooled results. Through further subgroup analysis, we found that a study from the American was the main cause of heterogeneity. As we removed the American study, we found that high expression of DLL3 is a marker of poor prognosis in SCLC. But we also noticed that the results of the American study were contrary to our conclusions, they reported that high expression of DLL3 is a marker of good prognosis in SCLC. One interpretation of this result was that DLL3 expression varies between different populations. The American study indicated that the high expression of DLL3 was a marker of good prognosis [21], and there was no significant correlation between DLL3 expression and prognosis in several Japan studies [22, 25], while the high expression of DLL3 is a marker of poor prognosis in China studies [20, 23]. These results showed that the expression of DLL3 might be different in different populations. However, only one study researched the correlation with DLL3 expression and prognosis in SCLC outside of Asia, so the results need to be treated with caution. In addition, more studies were suggested for the future to further verify the existence of such differences.

Our other explanation of this result was that the expression of DLL3 was the same in different populations. Among the included studies, the sample size of 5 studies was less than 100, including the America and Japan studies. Therefore, we speculated that insufficient sample size might cause bias in the results. We conducted a subgroup analysis according to the sample size, and the results showed that the high expression of DLL3 in the studies with large sample sizes (*N* ≥ 100) was associated with poor prognosis, while the pooled results of the studies with low sample sizes (*N* < 100) showed no significant correlation between the expression of DLL3 and prognosis. Therefore, the sample size may be one of the reasons for the differences in DLL3 expression in each study. Moreover, immunohistochemistry was used to detect DLL3 expression in all of the studies. Immunohistochemical staining is a semi-quantitative method and is evaluated with great subjectivity [28]. Different antibodies and different cut-off values for DLL3 expression were employed in all the included studies and thus could also be another cause of the differences in results. The American study also explained their different results by claiming that many of the other studies were performed using mRNA expression instead of protein expression or used different cut-offs value of DLL3 expression [21].

Researches have reported that DLL3 is highly expressed in SCLC [29, 30], which suggested that DLL3 might promote the development of SCLC. Therefore, we also discussed the correlation between DLL3 expression and the clinical characteristics of patients with SCLC. The pooled results showed that DLL3 expression had no significant correlation with patients’ sex, smoking status and stage, while DLL3 often highly expressed in metastasis patients of SCLC. Our survival analysis outcomes were consistent with this result, which suggested that high expression of DLL3 might be one of the factors contributing to poor prognosis in patients with advanced metastatic SCLC in Asia. However, only a few studies reported the correlation between the expression of DLL3 and clinical characteristics, and the methods and cut-offs values used to detect the DLL3 expression in each study were not uniform. Therefore, more reliable studies are needed to further verify our outcomes. Our results suggest that it is valuable to further investigate the correlation between DLL3 and the clinical characteristics of patients with SCLC.

Our meta-analysis is the first to focus on the prognostic value of DLL3 in SCLC. The significance of this meta-analysis lies in providing a basic direction and evidence for further research into the mechanism of DLL3 in SCLC. For SCLC, Notch1 over expression could induce G1 cell cycle arrest [31]. Previous studies reported that DLL3 downregulated the Notch receptor expression, thereby the Notch signalling pathway was inhibited within the cell [32]. Therefore, high expression of DLL3 can promote the development of SCLC by inhibiting the Notch signalling pathway. Studies also have shown that the high expression of DLL3 may reduce the sensitivity of chemotherapy drugs [24]. These studies have demonstrated that DLL3 may be associated with the prognosis of SCLC and also consistent with our meta-analysis results. Thus, studies of the corresponding targeted drugs of DLL3 can effectively inhibit the expression of DLL3 and thus improve the survival of SCLC. Rovalpituzumab tesirine (Rova-T) is a new antibody-drug conjugate directed against DLL3 in SCLC [33]. A phase I trial found that patients with high DLL3 expression in SCLC showed a better response to Rova-T than those with a low DLL3 expression [34]. However, disappointingly, the phase III TAHOE trial has been stopped because the Rova-T group showed a worse OS compared to the control group [35]. But more clinical trials are recruiting participator to investigate Rova-T as maintenance therapy in advanced stage SCLC. The lack of progress with this drug does not prevent us from making a breakthrough with other similar drugs. Some researches found that the intra-tumoural and inter-tumoural distribution of DLL3 protein in SCLC is homogeneous [20], supporting the conclusion that biopsy specimens are a reliable source for DLL3 evaluation for targeted therapy. In addition, most studies have demonstrated that DLL3 is highly expressed in SCLC, while it is not or is less expressed in other types of lung cancer and normal tissues [36]. Therefore, the expression of DLL3 can be detected by biopsy as an indicator for diagnosis, predicting therapeutic efficacy and monitoring recurrence or metastasis of SCLC in the future.

Although our study fully explains the prognostic value of DLL3 in SCLC, our analysis still has several limitations. First, large heterogeneity was observed in the pooled results. This is explained by the observation that the evaluation criteria for the expression of DLL3 are particularly mixed, and there are no international standards for cut-offs values to determine the expression of DLL3. Thus, the scoring methods and cut-offs values of DLL3 should be unified to strengthen our conclusions. Otherwise, the detection method of DLL3 in most studies is mainly immunohistochemistry at present, which is a semi-quantitative, subjective and inaccurate detection method. Different studies show different prognostic values of DLL3. Therefore, we need other more precise detection methods to evaluate the expression of DLL3 in SCLC in the future.

Second, the therapy method is also a key limitation. The current studies only focus on tissue specimens from patients with SCLC after surgery or biopsy, and few studies reported the treatment methods in their researches. However, the prognostic value of DLL3 may lie in the therapeutic method. Therefore, every study should pay attention to the impact of patient treatment methods on prognosis in the future.

Third, some of the original studies did not report the data of HR and 95% CI. The HR and 95% CI results were measured from survival curves, an evaluation method with certain deviation and subjectivity, which might influence the authenticity of the results.

## Conclusion

In summary, our meta-analysis confirmed that high expression of DLL3 was a potential poor prognostic biomarker for SCLC in Asian populations; moreover, DLL3 expression was correlated with advanced metastasis SCLC in Asian populations. However, the relationship between DLL3 expression and the prognostic or clinical characteristics of patients with SCLC in European and American populations need to be further verified. Thus, detecting the expression of DLL3 in tumour tissue will be helpful to guide therapy in Asian patients of SCLC. For our research, other high-quality studies, especially from European and American countries, are required to confirm our findings about the prognosis value of DLL3 in SCLC in the future. In view of the limitations of our analysis, the conclusions should be interpreted with caution.

## Supplementary information

**Additional file 1.** The PRISMA checklist.

**Additional file 2: Fig.A.1.** Forest plots of prognostic value of DLL3 in SCLC. SCLC(small cell lung cancer), HR(hazard ratios), 95% CI (95% confidence intervals), DLL3(delta-like protein 3), *I*^2^(percentage heterogeneity between studies), *p*(test for heterogeneity. **Fig.A.2.** Forest plots of the correlation between DLL3 expression and sex of patients with SCLC. SCLC(small cell lung cancer), RR(relative ratios), 95% CI (95% confidence intervals), DLL3(delta-like protein 3), *I*^2^(percentage heterogeneity between studies), *p*(test for heterogeneity). **Fig.A.3.** Forest plots of the correlation between DLL3 expression and smoking history of patients with SCLC. SCLC(small cell lung cancer), RR(relative ratios), 95% CI (95% confidence intervals), DLL3(delta-like protein 3), *I*^2^(percentage heterogeneity between studies), *p*(test for heterogeneity). **Fig.A.4.** Forest plots of the correlation between DLL3 expression and tumour stage of patients with SCLC. SCLC(small cell lung cancer), RR(relative ratios), 95% CI (95% confidence intervals), DLL3(delta-like protein 3), *I*^2^(percentage heterogeneity between studies), *p*(test for heterogeneity). **Fig.A.5.** Forest plots of the correlation between DLL3 expression and metastasis of patients with SCLC. SCLC(small cell lung cancer), RR(relative ratios), 95% CI (95% confidence intervals), DLL3(delta-like protein 3), *I*^2^(percentage heterogeneity between studies), *p*(test for heterogeneity). **Fig.A.6.** Sensitivity analysis of all the studies.

## Data Availability

The datasets used and/or analysed during the current study are available from the corresponding author on reasonable request.

## Abbreviations

CI: Confidence intervals
DLL3: Delta-like protein 3
DFS: Disease-free survival
DSS: Disease-specific survival
HR: Hazard ratios
IHC: Immunohistochemistry
OS: Overall survival
PFS: Progression-free survival
QUIPS: Quality In Prognosis Studies
RFS: Relapse/recurrence-free survival
RR: Risk ratio
SCLC: Small cell lung cancer
