# Supplementary material for: Potential prognostic value of delta-like protein 3 in small cell lung cancer: a meta-analysis
Source: World J Surg Oncol. 2020 Aug 26;18:226. doi: 10.1186/s12957-020-02004-5 (PMC7448456; doi:10.1186/s12957-020-02004-5)

Fig.A.1. Forest plots of prognostic value of DLL3 in SCLC. SCLC(small cell lung cancer), HR(hazard ratios), 95% CI (95% confidence intervals), DLL3(delta-like protein 3), I^2(percentage heterogeneity between studies), p(test for heterogeneity) .

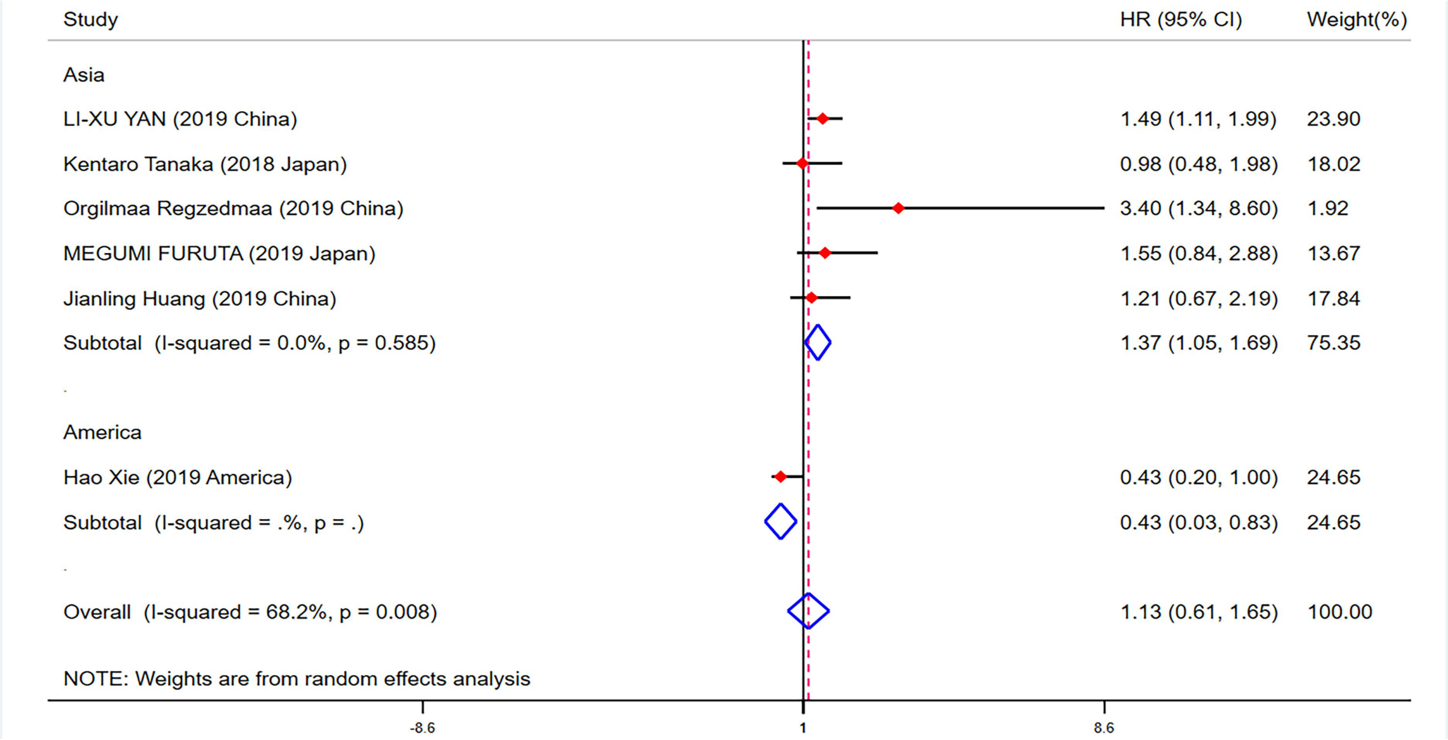

Fig.A.2. Forest plots of the correlation between DLL3 expression and sex of patients with SCLC. SCLC(small cell lung cancer), RR(relative ratios), 95% CI (95% confidence intervals), DLL3(delta-like protein 3), I^2(percentage heterogeneity between studies), *p*(test for heterogeneity) .

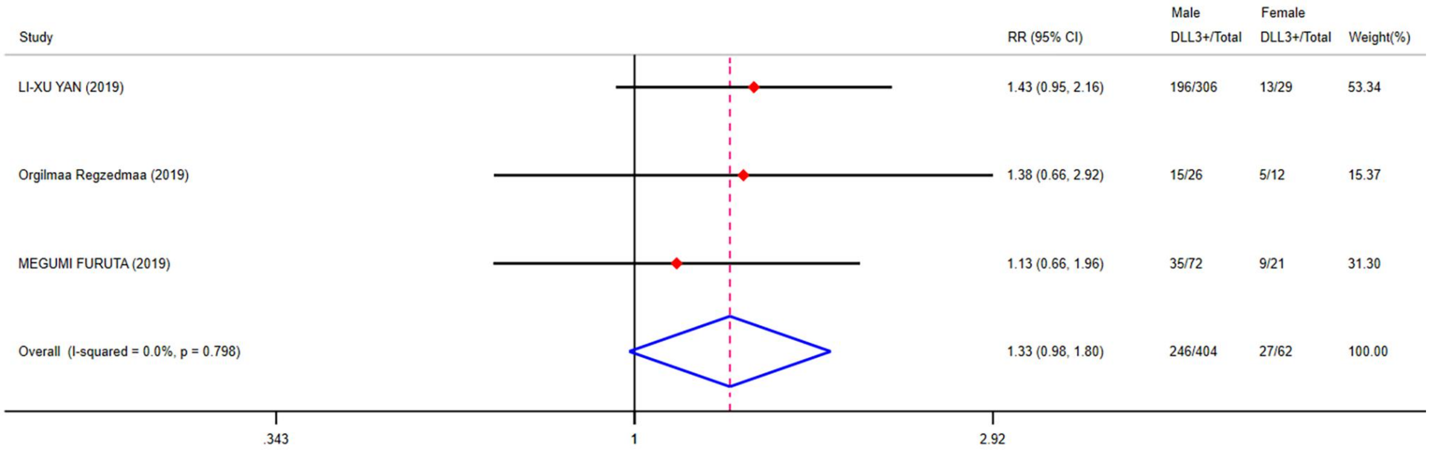

Fig.A.3. Forest plots of the correlation between DLL3 expression and smoking history of patients with SCLC. SCLC(small cell lung cancer), RR(relative ratios), 95% CI (95% confidence intervals), DLL3(delta-like protein 3), I^2(percentage heterogeneity between studies), *p*(test for heterogeneity) .

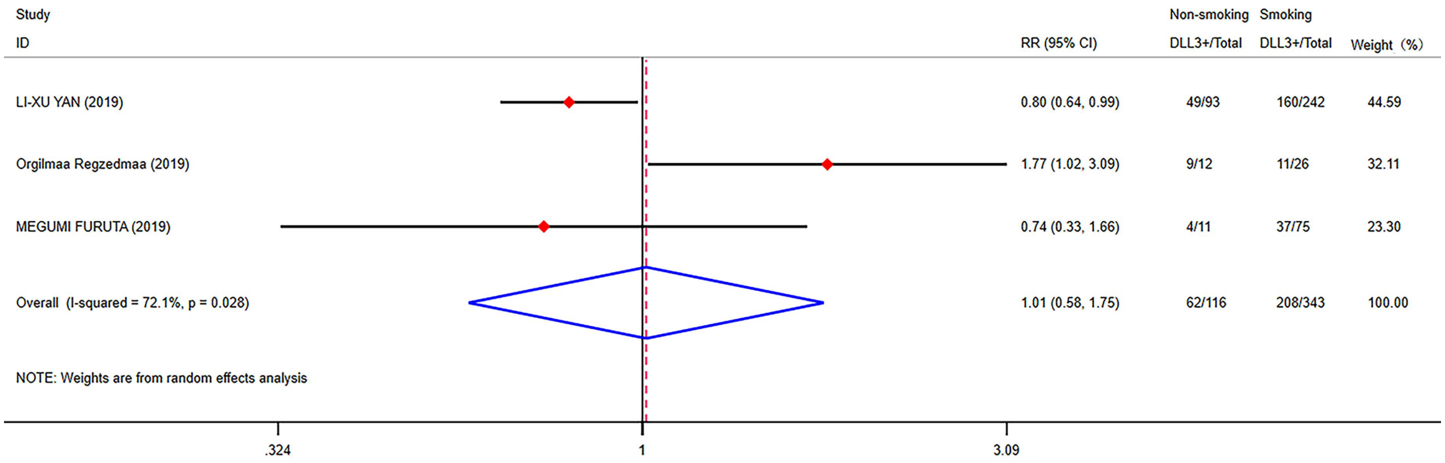

Fig.A.4. Forest plots of the correlation between DLL3 expression and tumour stage of patients with SCLC. SCLC(small cell lung cancer), RR(relative ratios), 95% CI (95% confidence intervals), DLL3(delta-like protein 3), I^2(percentage heterogeneity between studies), p(test for heterogeneity) .

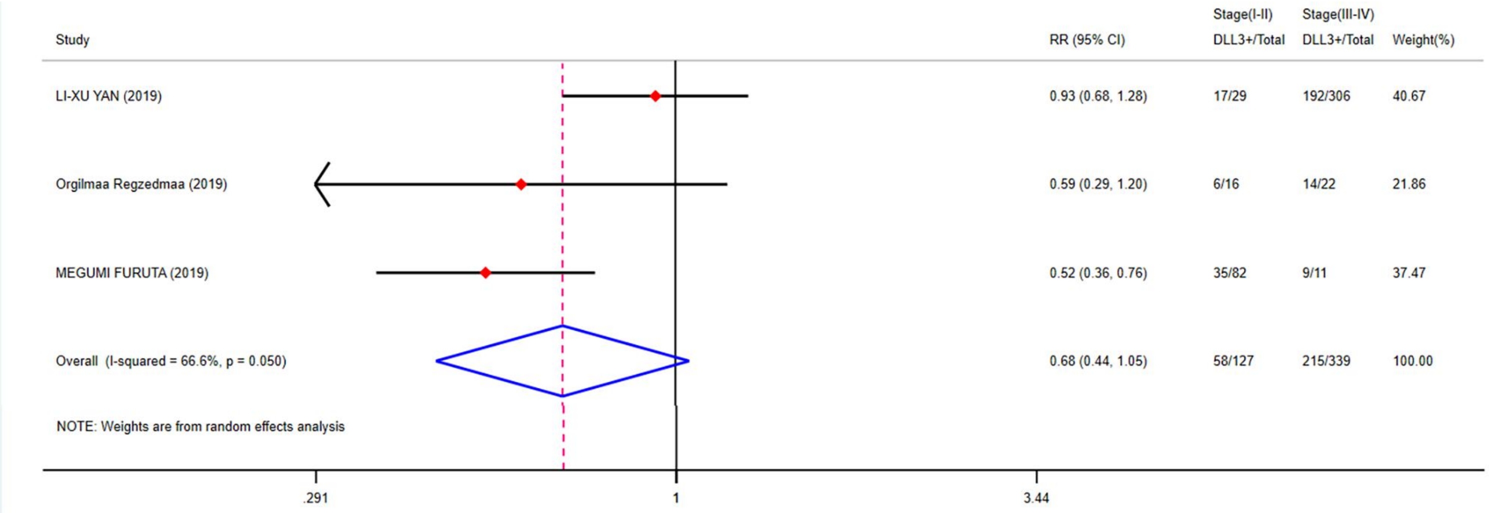

Fig.A.5. Forest plots of the correlation between DLL3 expression and metastasis of patients with SCLC. SCLC(small cell lung cancer), RR(relative ratios), 95% CI (95% confidence intervals), DLL3(delta-like protein 3), I^2(percentage heterogeneity between studies), p(test for heterogeneity) .

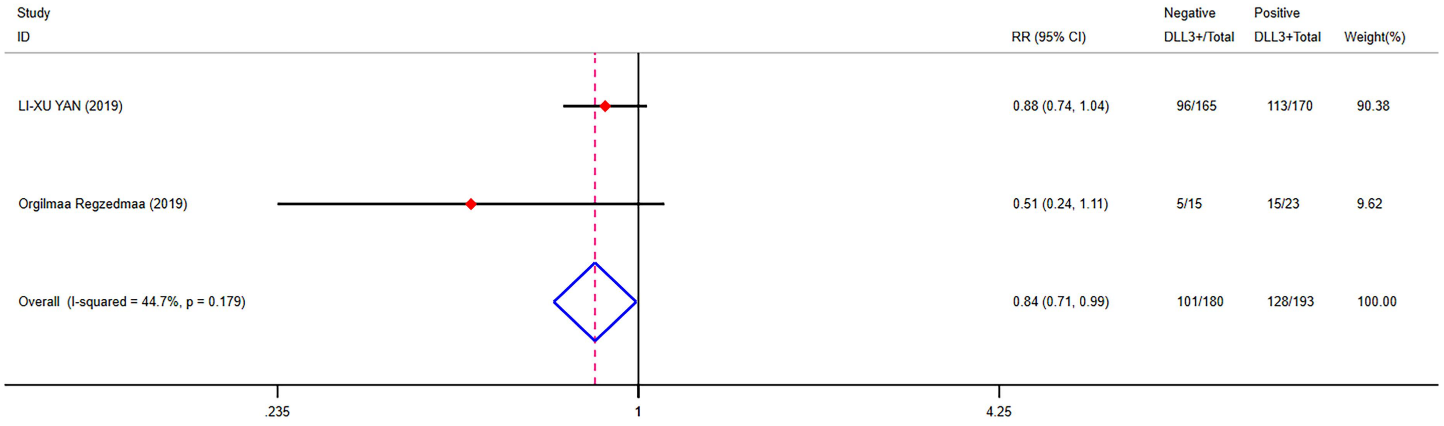

Fig.A.6. Sensitivity analysis of all the studies.

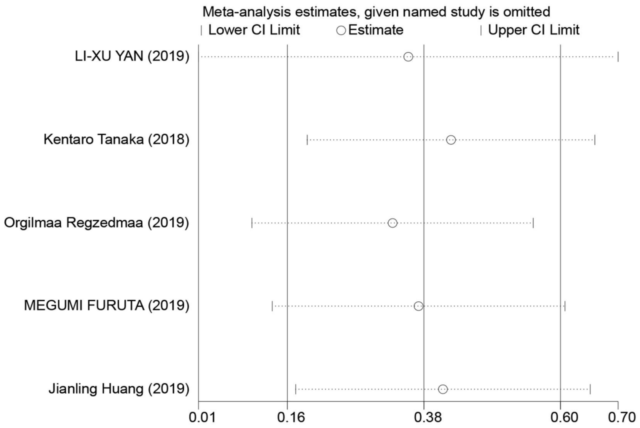

Supplement: Supplementary file 2 — Additional file 2: Fig.A.1. Forest plots of prognostic value of DLL3 in SCLC. SCLC(small cell lung cancer), HR(hazard ratios), 95% CI (95% confidence intervals), DLL3(delta-like protein 3), I2(percentage heterogeneity between studies), p(test for heterogeneity. Fig.A.2. Forest plots of the correlation between DLL3 expression and sex of patients with SCLC. SCLC(small cell lung cancer), RR(relative ratios), 95% CI (95% confidence intervals), DLL3(delta-like protein 3), I2(percentage heterogeneity between studies), p(test for heterogeneity). Fig.A.3. Forest plots of the correlation between DLL3 expression and smoking history of patients with SCLC. SCLC(small cell lung cancer), RR(relative ratios), 95% CI (95% confidence intervals), DLL3(delta-like protein 3), I2(percentage heterogeneity between studies), p(test for heterogeneity). Fig.A.4. Forest plots of the correlation between DLL3 expression and tumour stage of patients with SCLC. SCLC(small cell lung cancer), RR(relative ratios), 95% CI (95% confidence intervals), DLL3(delta-like protein 3), I2(percentage heterogeneity between studies), p(test for heterogeneity). Fig.A.5. Forest plots of the correlation between DLL3 expression and metastasis of patients with SCLC. SCLC(small cell lung cancer), RR(relative ratios), 95% CI (95% confidence intervals), DLL3(delta-like protein 3), I2(percentage heterogeneity between studies), p(test for heterogeneity). Fig.A.6. Sensitivity analysis of all the studies. [file 12957_2020_2004_MOESM2_ESM.pdf]
